# The Modulation of Endoplasmic Reticulum Stress by Chemical Chaperone Upregulates Immune Negative Cytokine IL-35 in Apolipoprotein E-Deficient Mice

**DOI:** 10.1371/journal.pone.0087787

**Published:** 2014-01-30

**Authors:** Bo Wang, Shen Dai, Zhaojing Dong, Yue Sun, Xingguo Song, Chun Guo, Faliang Zhu, Qun Wang, Lining Zhang

**Affiliations:** Department of Immunology, Shandong University School of Medicine, Jinan, Shandong, China; University of Manitoba, Canada

## Abstract

Interleukin (IL)-35 is a newly identified immune negative molecule which is secreted by CD4^+^Foxp3^+^ T regulatory cells (Tregs) and contributes to their suppressive capacity. Early data have shown that IL-35 inhibits development of several autoimmune diseases. However, the role of IL-35 in atherosclerosis, a lipid-driven chronic inflammatory disease in arterial wall, remains to be investigated. Here, we found that IL-35 was involved in atherosclerosis in apolipoprotein E-deficient (ApoE^−/−^) mice. ApoE^−/−^ mice with established atherosclerotic lesion displayed a lower level of IL-35 compared to age-matched wild type C57BL/6 mice without plaque. However, IL-35 expression increased significantly in ApoE^−/−^ mice with attenuated plaque. More importantly, we found that modulation of ER stress treated by chemical chaperone, 4-Phenyl butyric acid (PBA) *in vivo*, mainly upregulated immune negative regulating molecule IL-35, as well as IL-10 and Foxp3, accompanied by increased Tregs. However, no obvious impact on pro-inflammatory molecules such as TNF-α, IFN-γ, IL-17 and IL-23 was observed, which provides new insight into the benefit of ER stress recovery from attenuated plaque. Our results suggest that IL-35 might have a potential value for atherosclerotic therapy.

## Introduction

Interleukin (IL)-35 is a newly identified inhibitory cytokine produced by CD4^+^Foxp3^+^ T regulatory cells (Tregs) and is required for suppressive function of Tregs [1], [2]. IL-35 belongs to the IL-12 family in which all members including IL-12, IL-23, IL-27 as well as IL-35 exist as heterodimers that are composed of an α chain (p19, p28, p35) and a β chain (p40 and Epstein-Barr virus induced gene 3, EBI3). IL-35 (p35 and EBI3) shares p35 with IL-12 (p35 and p40) and EBI3 with IL-27 (p28 and EBI3) [3], [4]. On one hand, IL-35 is able to promote the expansion of Tregs and suppress the proliferation of conventional T cells (Tconv cells) and differentiation of Th1 and Th17 *in vitro* and *in vivo* [5], [6]. On the other hand, IL-35 can converse naive T cells into strongly suppressive Treg cells called iTr35 cells, which also function via IL-35 [7]. IL-35 has been shown to inhibit several inflammatory disorders, including inflammatory bowel disorder [8], autoimmune encephalomyelitis [9], autoimmune diabetes [10], collagen II-induced arthritis [11], and airway inflammation [12]. However, the role of IL-35 in atherosclerosis, a lipid-driven chronic inflammation of the walls of large and medium-sized arteries, remains to be investigated. It has been reported that Tregs play an inhibitory effect on development of atherosclerosis [13∼16], and the level of plasma IL-35 decreases in patients with acute coronary syndrome (unstable angina pectoris and acute myocardial infarction, AMI) compared with chest pain syndrome group [17]. Two subunits of IL-35, EBI3 and p35, are also strongly expressed in human advanced plaque [18], suggesting that IL-35 may be involved in atherosclerosis.

Endoplasmic reticulum (ER) is an organelle in which newly synthesized proteins are correctly folded and assembled. Once it is perturbed by various pathological conditions, unfolded proteins will accumulate and result in endoplasmic reticulum stress (ER stress), also defined as the unfolded protein response (UPR). Accumulating evidence indicates that the UPR is chronically activated in atherosclerotic lesions by oxidative stress, high levels of intracellular cholesterol and saturated fatty acids [19], [20]. The prolonged activation of the UPR further triggers cell apoptosis in atherosclerosis [21], [22]. In addition to apoptosis, ER stress also mediates inflammation that contributes to the development of atherosclerosis [23∼25]. Furthermore, recovery of ER function is believed to be a critical factor for improvement of atherosclerosis [26]. 4-Phenylbutyric acid (PBA), a chemical chaperone, has been shown to attenuate atherosclerotic lesions through improvement of ER function *in vivo* [27].

The subunits of IL-35, p35 and EBI3, are synthesized and assembled in ER [28], [29], and it is supposed that modulation of ER stress by PBA can affect production of IL-35. In the present study, we investigated the possible role of IL-35 in atherosclerosis in apolipoprotein E-deficient (ApoE^−/−^) mice fed on a high-fat diet with or without PBA treatment.

## Materials and Methods

### Induction of Atherosclerosis

Male C57BL/6 wild type mice and male ApoE^−/−^ mice were purchased from Beijing University and fed on a high-fat diet (0.25% cholesterol and 15% cocoa butter) from 8 weeks of age to 16 weeks to induce atherosclerotic plaques. All animal studies were approved by the Animal Care and Utilization Committee of Shandong University, China.

### Application of PBA *in vivo*

ApoE^−/−^ mice (n = 10 for each group) were fed on a high-fat diet (0.25% cholesterol and 15% cocoa butter) from 8 weeks of age. Two weeks after high-fat diet, mice were divided into two groups randomly, one group, named as PBA, was injected intraperitoneally with PBA (P21005, Sigma-Aldrich, St. Louis, MO, USA) in phosphate buffered saline (PBS), 100 mg/kg, one time per three days for 5 weeks. The other group was injected intraperitoneally with PBS as control. Mice were sacrificed for analysis one week after the last treatment.

### Metabolic Studies

Mice were weighed at the end of experiment. Total plasma cholesterol (TCH), total triglycerides (TGs) and high-density lipoprotein (HDL) levels were determined with automated enzymatic technique (7080, HI TACH, Japan). Low-density lipoprotein (LDL) was detected with an automated chemically modified technique (Roche Modular DPP System, Roche, Switzerland).

### Western Blots

Proteins obtained from separate thoracic and abdominal aorta, were separated by SDS-PAGE and blotted on PVDF membranes. Membranes were probed with primary antibodies, Phospho-eIF2a (p-eIF2a) (#3597 Cell Signaling Technology, USA), eIF2a (#9722 Cell Signaling Technology, USA), spliced XBP-1 (sXBP-1) (ab37152, Abcam, Hong Kong) and β-actin (Bioworld, Georgia, USA) overnight at 4°C, respectively, followed by secondary antibody conjugated with peroxidase (A0208, Beyotime, China) for one hour at room temperature. After washing, signals were visualized using electrochemiluminescence (Pierce Biotechnology, Rockford, IL, USA) and autoradiography.

### Immunohistochemistry and Immunofluorescence

After sacrificing, mice were perfused with PBS through the left ventricle and hearts were fixed in 4% paraformaldehyde solution overnight and embedded in optimal cutting temperature (OCT) (Sakura Finetek, Torrance, CA, USA) medium or paraffin. Aortic root sections were cut from the embedded hearts. Serial cryosection of 6∼10 µm or paraffin-section of 4∼6 µm were dissected along the longitudinal direction of aortic root vessels using cryotome (HM550, Thermo scientific, USA). Five sections spaced 80 µm apart of each aorta root were stained with oil red “O” staining (O0625, Sigma-Aldrich, USA). Corresponding frozen sections were subjected to immunohistochemical staining according to respective antibody protocols, including rat anti-mouse macrophage Moma-2 (MCA519A647, AbD serotec, Oxford, U.K.), rabbit polyclonal to α- smooth muscle actin (ab5694, Abcam, Hong Kong), rat anti-mouse CD3 (MCA500G, AbD Serotec, Oxford, U.K.), rabbit anti-mouse Foxp3 (BA2032-1, BOSTER, China) and rabbit anti Phospho-PERK (p-PERK, 3179, Cell Signaling Technology, Danvers, MA, USA) to detect the levels of macrophage, smooth muscle cell, CD3^+^ T cell, Foxp3^+^ cells and p-PERK in lesion respectively. Alexa Fluor 555-labeled goat anti-rabbit IgG (A0462. Beyotime, China) was used to perform indirect immunofluorescence assays of Foxp3. Terminal deoxynucleotidyl transferase mediated dUTP nick end labeling (TUNEL) was performed on paraffin sections with TUNEL kit (Roche, USA) according to manufacturer’s instructions. Images were captured using an Olympus microscope (IX71; Olympus Corporation, Tokyo, Japan). The area of plaque and positive staining were measured using Image-Pro Plus software (Media Cybernetics, Bethesda, MD, USA).

### RNA Isolation and Real-Time PCR

Total RNA was isolated from thoracic and abdominal aorta using TRIzol reagent (15596-026, Invitrogen, Carlsbad, California, USA). Reverse transcription was performed with RT-PCR quick master mix (PCR-311, TOYOBO, Japan) to get cDNA, and real-time quantitative polymerase chain reactions were performed with Ultra SYBR mixture (CW0956, CW Bio, China) using CFX 96 Real-Time Detection System (Bio-RAD, USA). Sequences of related gene specific primers were included in Table 1. Data of relative molecule expression was presented by real-time quantitative PCR using the ΔΔCt model, and our data are reported as the fold change in the experimental group normalized to an endogenous reference gene (ACTB) and relative to the control group.

**Table 1:** Summary of the primers for real time-PCR.

Genes	Primers	Sequences
IFN-γ	Forward	CGGCACAGTCATTGAAAGCCTA
	Reverse	GTTGCTGATGGCCTGATTGTC
TNF-α	Forward	CCCTCACACTCAGATCATCTTCT
	Reverse	TGCTACGACGTGGGCTACAG
IL-17A	Forward	CTGATCAGGACGCGCAAAC
	Reverse	TCGCTGCTGCCTTCACTGTA
IL-23	Forward	GGACTTGTGCTGTTCTTGTTTTGT
	Reverse	CCTGCTCTGGGGTTTGTTTC
TGF-β	Forward	GTGTGGAGCAACATGTGGAACTCTA
	Reverse	CGCTGAATCGAAAGCCCTGTA
IL-10	Forward	GCCAGAGCCACATGCTCCTA
	Reverse	GATAAGGCTTGGCAACCCAAGTAA
EBI3	Forward	AGCAGCCTCCTAGCCTTTGTGG
	Reverse	GAGTTCCTGAGGGTGAAAGTCGTG
IL-12α	Forward	AGCGTTCCAACAGCCTCAC
	Reverse	CTCTGGCCGTCTTCACCAT
IL-12β	Forward	TGTCACCAGCAGTTGGTCATCTC
	Reverse	CTCACTGCTCTGGTCCAAGGTC
p28	Forward	GCACAGGCACCTCCGCTTTCA
	Reverse	GCAGCAGCAGGTCCCGAACAG
Foxp3	Forward	CCCAGGAAAGACAGCAACCTT
	Reverse	TTCTCACAACCAGGCCACTTG

### Flow Cytometry

Splenocytes isolated from PBA-treated and control mice were washed with PBS and stained with PE-Cy5-conjugated anti-CD4 (Anti-Mouse CD4 PE-Cyanine5, eBioscience, San Diego, CA, USA) and then were fixed and perforated by the fixation and permeabilization kit (eBioscience, San Diego, CA, USA) and then stained intracellularly with PE-conjugated anti-Foxp3 monoclonal antibodies (Anti-Foxp3 PE, eBioscience, San Diego, CA, USA). Flow cytometric analysis was performed using a Cytomics FC500 (Beckman Coulter, Brea, CA, USA) and the data were analyzed by CXP2.0 software.

### Detection of Cytokines

Serum was collected for detection of TNF-α, IFN-γ, IL-17 and IL-10 using a mouse cytometric bead array (Cytometric Bead Array, BD Biosciences, San Jose, CA, USA) and for assays of IL-23 (EMC114.48, NeoBioscience, China), and TGF-β (BMS608/4, eBioscience, San Diego, CA, USA) using ELISA according to manufacturer’s instructions. IL-35 in serum was quantified using IL-35 ELISA kits (RapidBio Lab, USA) according to the manufacturer's instruction and the sensitivity of this assay is ≥1.0 pg/ml.

### Statistics

All analyses were done by SPSS 11.0 (SPSS, Chicago, IL,USA). Data are expressed as mean ± SEM. Unpaired *t* tests were used for comparison of different group data. P value <0.05 was considered as significant.

## Results

### The Modulation of ER Stress and Attenuation of Atherosclerotic Lesion in ApoE^−/−^ Mice Treated by PBA

**Figure 1:**
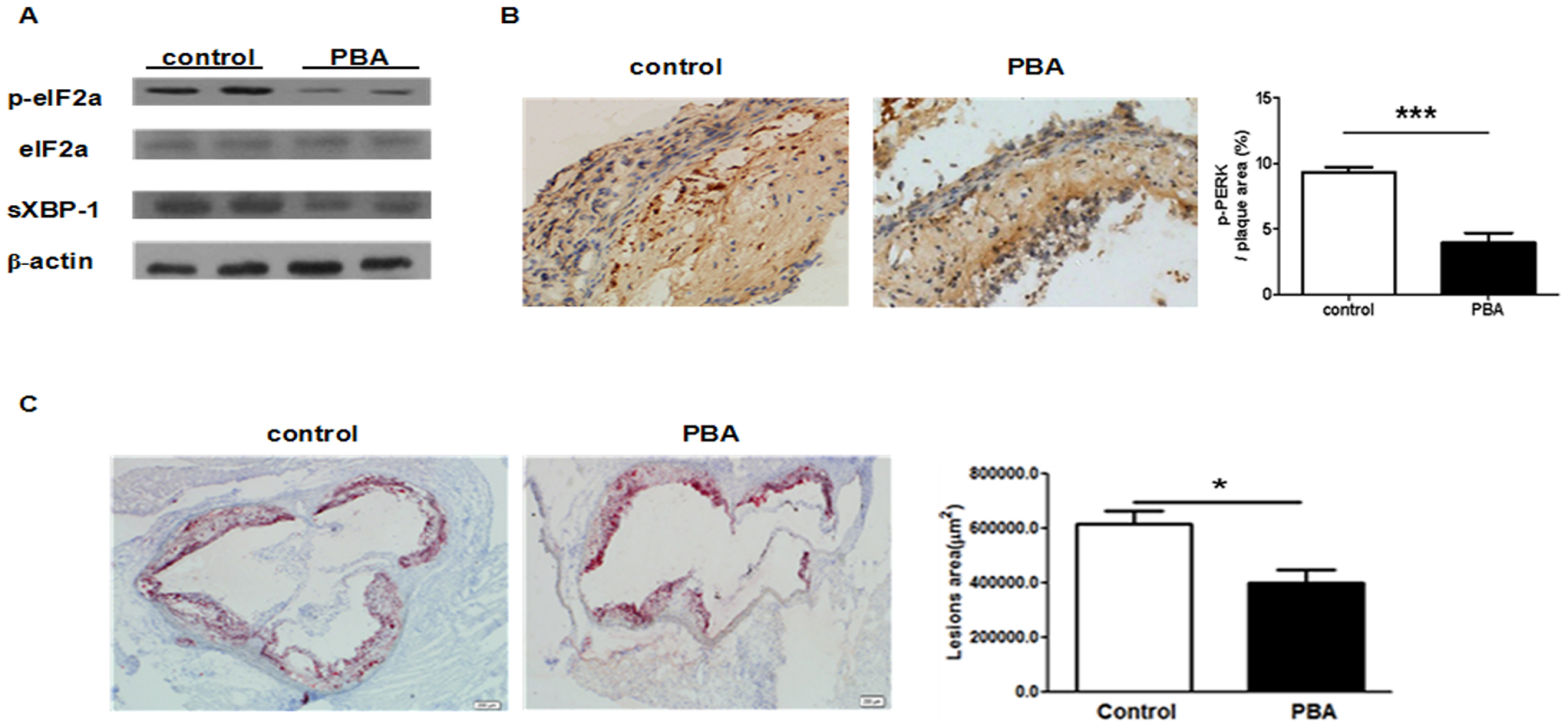
PBA treatment inhibited ER stress and ameliorated atherosclerosis *in vivo*. **A.** Protein was obtained from the thoracic and abdominal aorta of ApoE^−/−^ mice with or without PBA treatment and the expressions of phosphorylated eIF-2α (p-eIF2α), total eIF-2α, spliced XBP-1 (sXBP-1) and β-actin were detected by Western Blot. (n = 2 per group) **B.** The aortic root sections were stained by rabbit anti- phosphorylated-PERK (p-PERK) and expression of p-PERK in plaque was analyzed by immunohistochemistry. (n = 10 per group), *p<0.05, ***p<0.001.

**Figure 2:**
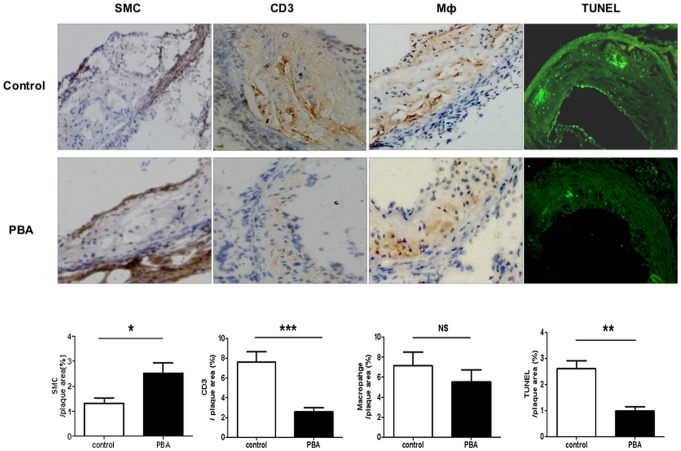
PBA treatment influenced the stability of plaque. Representative photomicrographs and the quantitative analysis of smooth muscle cell (SMC), CD3, macrophage (Mφ), and TUNEL in lesion of aortic root of ApoE^−/−^ mice. The aortic root sections were stained with rabbit polyclonal to α- smooth muscle actin, rat anti-mouse macrophage Moma-2 and rat anti mouse CD3. The content of macrophage, smooth muscle cell, CD3 T cell in lesion was analyzed respectively by immunohistochemistry. For detection of cell apoptosis, sections were incubated with anti-TUNEL antibody and the content of apoptotic cells was analyzed by immunofluorescence. (n = 10 per group), *p<0.05, **p<0.01, ***p<0.001.

Previous researches have demonstrated that ER stress is involved in atherosclerosis and recovery of ER function is believed to be a critical factor for improvement of atherosclerosis [30]–[33]. Here, we firstly observed the impact of ER stress improvement by PBA treatment on atherosclerotic lesion. Consistent with previous research [27], the expression of ER stress signal proteins, p-eIF2α, sXBP-1 detected by western blots (Fig. 1A) and p-PERK by immunohistochemistry (Fig. 1B) were significantly decreased in mice treated with PBA compared to the control. Subsequently, PBA injection led to a decrease in plaque area in aortic root (Fig. 1C). Furthermore, the number of smooth muscle cells (SMC) increased while CD3^+^ T cells and TUNEL positive apoptotic cells (Fig. 2) in lesion decreased in the PBA injection group compared to the control. In addition, as shown in Table 2, there were no significant differences in body weight and plasma lipid profile between the PBA-treated group and the control. This indicates that modulation of ER stress by PBA attenuates the formation of lesion and increases the stability of plaque.

**Table 2:** PBA treatment did not change body weight and lipid profile.

	Weight (g)	TG (mmol/L)	TCH (mmol/L)	LDL (mmol/L)	HDL (mmol/L)
Control	30.26±0.85	47.74±1.16	4.33±1.24	7.72±0.17	11.25±1.26
PBA	33.10±1.60	46.51±1.39	3.93±0.96	7.65±0.29	10.49±2.44
P value	0.17	0.52	0.82	0.82	0.79

The data are given as the mean**±**SD. TG:total triglycerides; TCH:total cholesterol; LDL: low-density lipoprotein cholesterol; HDL: high-density lipoprotein cholesterol.

### The Increase of IL-35 in ER Stress-attenuated Lesion

**Figure 3:**
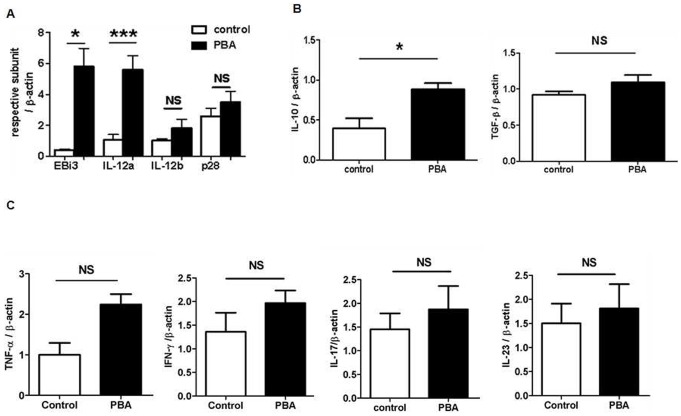
The influence of PBA treatment on cytokines in arterial wall. The expression of cytokine mRNAs in the thoracic and abdominal aorta was quantified by real -time PCR analysis and normalized to β-actin. Fold-changes in expression in PBA treated mice relative to controls are shown. **A.** IL-35-related four subunits, EBI3, IL-12a, IL-12b and p28. **B.** IL-10 and TGF-β. **C. TNF-α,** IFN-γ, IL-17 and **IL-23**. (n = 6 per group). *p<0.05, **p<0.01, ***p<0.001.

We further investigated the change of cytokines in lesion after ER stress modulation by real-time PCR. As shown in Figure 3A, the recovery of ER stress by PBA significantly upregulated the expression of two IL-35-related subunits, IL-12α (p35) and EBI3, but had no effect on other subunits, IL-12β and p28, implying an increase in the level of IL-35 after modulation of ER stress. Meanwhile, the modulation of ER stress also increased IL-10 level but had no effect on the level of TGF-β (Fig. 3B) and pro-inflammatory cytokines, TNF-α, IFN-γ, IL-17 and IL-23 (Fig. 3C).

### The Increase of Serum IL-35 in ApoE^−/−^ Mice after PBA Treatment

**Figure 4:**
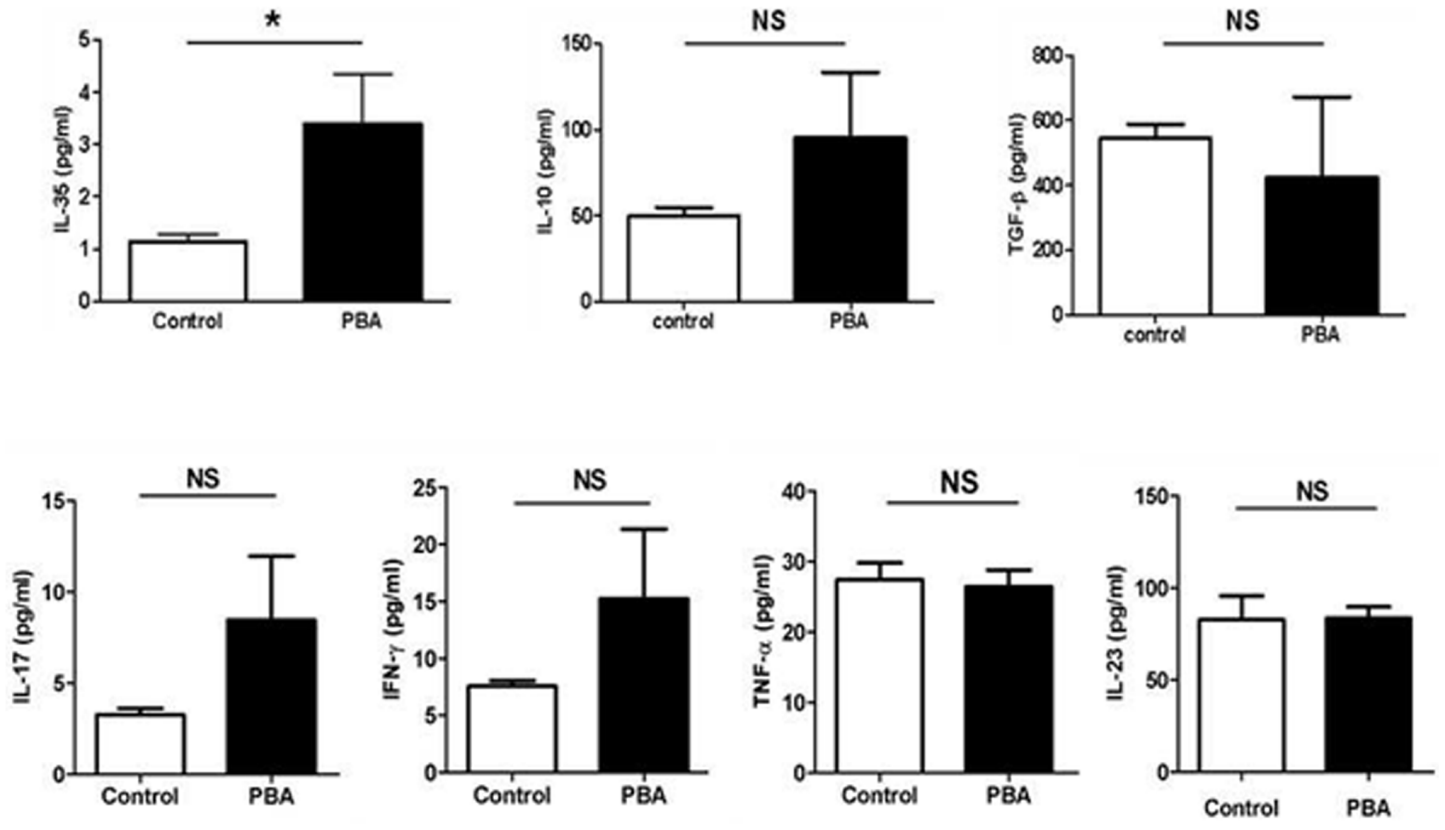
Impact of PBA treatment on circulating cytokines. Serum was obtained from ApoE^−/−^ mice with or without PBA treatment and the concentrations of **IL-23**, TGF-β and IL-35 in serum were detected by ELISA.The concentrations of **TNF-α**, IFN-γ, IL-17 and IL-10 were analyzed by cytometric bead array. (n = 6 per group),*p<0.05.

The change in circulating cytokines was detected by cytometric bead array for TNF-α, IFN-γ, IL-17 and IL-10, and by ELISA for IL-23, TGF-β and IL-35. As shown in Figure 4, PBA treatment significantly increased the level of serum IL-35. In addition, the level of IL-10 showed an increasing potential. However, consistent with our findings in lesion, PBA treatment had no effect on other cytokines including TGF-β, TNF-α, IFN-γ, IL-17 and IL-23.

### Upregulation of Foxp3^+^ Tregs in ApoE^−/−^ Mice after PBA Treatment

**Figure 5:**
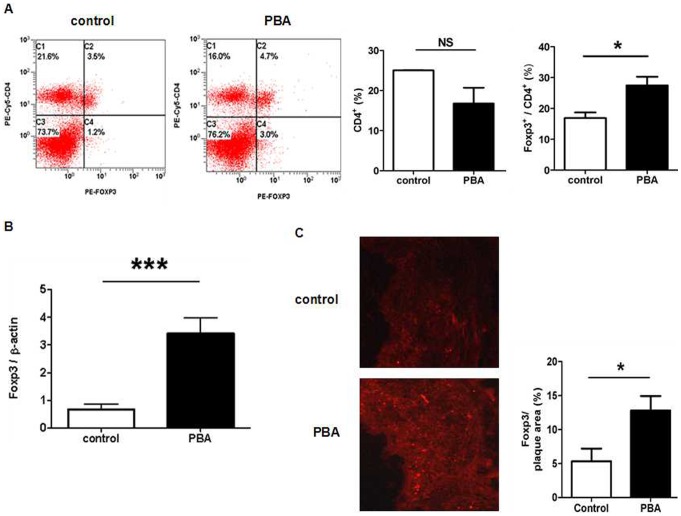
PBA treatment impacted Treg cells. **A.** Spleen cells were obtained from ApoE^−/−^ mice with or without PBA treatment and percentage of CD4+ T cells in spleen cells and percentage of CD4+Foxp3+ Tregs in total CD4+ T cells was analyzed by flow cytometry. **B.** The expression of Foxp3 mRNAs in the thoracic and abdominal aorta was quantified by real time-PCR analysis. Normalized to β-actin, fold-changes in expression in PBA treated mice relative to controls are shown. **C**. The expression of Foxp3 in plaque was analyzed by immunohistochemistry and immunofluorescence. The bright red dots in pictures are Foxp3^+^ cells (n = 6 per group). *P<0.05, ***p<0.001.

It has been reported that IL-35 is not only an effector molecule of Tregs but also an inducer of Tregs. Therefore, we detected the effect of PBA treatment on Treg cells. The spleen cells were collected from PBA treatment or control group and stained with CD4 and Foxp3 specific antibodies, and then the proportion of Tregs (CD4^+^ Foxp3^+^) in CD4^+^ T cells was analyzed by Flow Cytometry. As shown in Figure 5A, after PBA treatment, the proportion of total CD4^+^ T cells in spleen showed no significant change but the percentage of Foxp3^+^ Tregs in CD4^+^ T cells markedly increased compared with the control. We further measured the mRNA level of Foxp3, a specific transcriptional factor for Tregs in lesion. As shown in Figure 5B, PBA treatment led to an increase of Foxp3 mRNA in local plaque. Immunohistochemical staining of Foxp3 confirmed the result (Fig. 5C). Collectively, the modulation of ER stress increases the Foxp3^+^ Tregs both in lesion and peripheral immune organ.

### The Low Expression of IL-35 in ApoE^−/−^ Mice with Atherosclerotic Plaque

**Figure 6:**
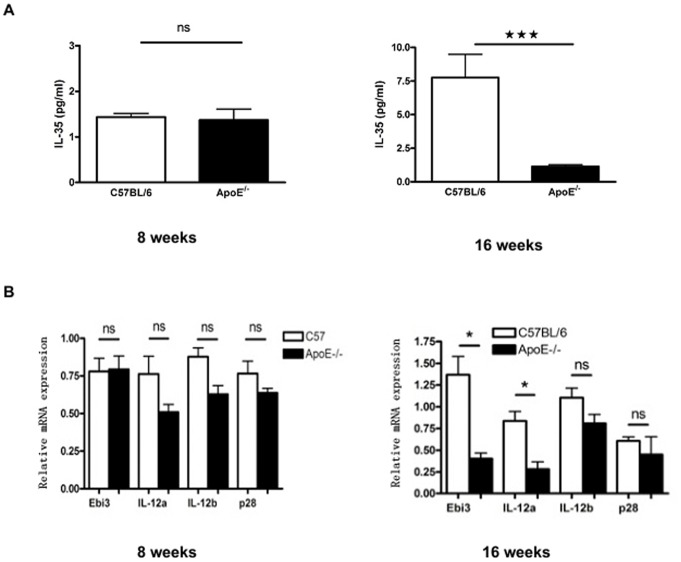
IL-35 expression in ApoE^−/−^ mice with atherosclerotic plaque. **A.** Serum was obtained from C57BL/6 and ApoE^−/−^ mice at 8 weeks of age fed with normal diet, without plaque (8 weeks) and at 16 weeks of age, after 8 weeks on a high-fat diet, C57BL/6 without plaque and ApoE^−/−^ mice with established plaque (16 weeks). The concentrations of IL-35 (pg/mL) in serum were detected by ELISA. **B.** Total RNA was isolated from thoracic and abdominal aorta of C57BL/6 and ApoE^−/−^ mice at 8 weeks of age fed with normal diet, without plaque (8 weeks) and at 16 weeks of age, for 8 weeks after high-fat diet, C57BL/6 without plaque and ApoE^−/−^ mice with established plaque (16 weeks). The expression of IL-35-related subunits was analyzed by real-time PCR. (n = 3 per group), *p<0.05,***p<0.001.

The above results indicate that modulation of ER stress by PBA attenuates atherosclerotic lesion and upregulates level of IL-35 both in arterial wall and in circulation. To further confirm the association of IL-35 with atherosclerosis, we detected the change of serum IL-35 level in development process of plaque in ApoE^−/−^ mice compared with wild type C57BL/6 mice. As shown in Figure 6A, there was a basic level of IL-35 in serum of wild type C57BL/6 as well as ApoE^−/−^ mice without plaque at 8 weeks. After 8 weeks high-fat diet, while C57BL/6 mice without plaque showed an increased IL-35 level, ApoE^−/−^ mice with established plaque remained low level of IL-35. The mRNA level of IL-35 subunits p35 and EBI3 in artery wall also decreased in ApoE^−/−^ mice with plaque, compared with wild type C57BL/6 (Fig. 6B). This suggests that the low level of IL-35 might be associated with formation of atherosclerosis lesion.

## Discussion

Here, we find that IL-35 is involved in atherosclerosis in ApoE^−/−^ mice. ApoE^−/−^ mice with atherosclerotic lesion have lower levels of IL-35 but the level increases in mice with attenuated plaque treated with PBA. More importantly, we find that modulation of ER stress by PBA treatment mainly upregulates immune negative regulating molecules, IL-35 as well as IL-10 and the transcription factor Foxp3, but has no obvious impact on pro-inflammatory molecules, such as TNF-α, IFN-γ, IL-17 and IL-23, which provides a new insight into the benefits of ER stress recovery to attenuated plaque.

To date, little is known about the role of IL-35 in atherosclerosis. Although previous research shows that expression of EBI3 and p35 is detected in human atheroma plaques, but other subunits, IL-27 α/p28 can also be found in plaque [34]. Therefore, it cannot be concluded that IL-35 exists in plaque. Here, we detected four subunits of IL-12 family, p35, p28, p40 and EBI3, and found that only two subunits of IL-35, p35 and EBI3, increased in arterial wall treated with PBA, but other related subunits, p40 (IL12β ) and p28 (IL-27 α), did not change. This suggests an increased level of IL-35 in attenuated plaque treated with PBA.

Atherosclerotic plaques, particularly advanced lesions, contain a large amount of toxic lipids (such as saturated fatty acids or free cholesterol) and pro-inflammatory cytokines (TNF-α, IFN-γ, IL-6, IL-12 etal). The pathophysiological environment can activate UPR which in turn upregulates expression of inflammatory genes (such as IFN-γ and IL-17) accelerating the progress of atherosclerotic plaque [35], [36]. Here, our results showed that modulation of ER stress by PBA application *in vivo* significantly decreased cell apoptosis, inflammation and size of plaque and increased the stability of plaque, which was consistent with previous research. Interestingly, the modulation of ER stress mainly upregulated immune negative regulating molecules such as IL-35, as well as IL-10 and Foxp3 in present research, but had no obvious impact on pro-inflammatory molecules, such as IL-17 and IFN-γ. However, the mechanism of this phenomenon is unknown. Previous research has shown that when overexpressed in cells, EBI3 tends to accumulate in an immature form in the ER associated with the molecular chaperone, calnexin, compatible with the notion that EBI3 associates with another partner that was not sufficiently abundant in these cells to enable its secretion [28], [29]. Therefore, one possible explanation is that PBA may help the assemblies of EBI3 with IL-12α (p35) and then result in an increased IL-35 which in turn amplifies Tregs or induces new Treg production, as supported by the elevation of IL-10 and Foxp3 expression in plaque and the increase of Foxp3^+^ Treg in spleen.

Although our research did not confirm the causal relationship of IL-35 with atherosclerosis absolutely, it provided a new direction for the research about atherosclerosis. Because IL-35 comprises two subunits, p35 and EBI3, which take part in the composition of IL-12 and IL-27 respectively, it is difficult to produce recombined IL-35 and neutralizing antibody or knockdown/knockout related genes without influencing other cytokines. We believe that further research should overcome those difficulties and the detailed mechanism of IL-35 influencing atherosclerosis would be elucidated in future.

Collectively, our results indicate that IL-35 is involved in the atherosclerosis. The modulation of ER stress by PBA is able to upregulate cytokine IL-35 which may contribute to attenuation of plaque in ApoE^−/−^ mice.

## References

[1] Li X, Mai J, Virtue A, Yin Y, Gong R (2012) etal (2012) IL-35 is a novel responsive anti-inflammatory cytokine–a new system of categorizing anti-inflammatory cytokines. PLoS One 7(3): e33628. 22438968 10.1371/journal.pone.0033628 PMC3306427

[2] Chaturvedi V, Collison LW, Guy CS, Workman CJ, Vignali DA (2011) Cutting edge: Human regulatory T cells require IL-35 to mediate suppression and infectious tolerance. J Immunol 186: 6661–6666. 21576509 10.4049/jimmunol.1100315 PMC3110563

[3] Ning-Wei Z (2010) Interleukin (IL)-35 is raising our expectations. Rev Med Chil 138: 758–66. 20919488 10.4067/s0034-98872010000600015

[4] L.W Collison, G.M Delgoffe, C.S Guy, K.M Vignali, V Chaturvedi (2012) etal (2012) The composition and signaling of the IL-35 receptor are unconventional. Nat Immunol 13: 290–299. 22306691 10.1038/ni.2227 PMC3529151

[5] Yang J, Yang M, Htut TM, Ouyang X, Hanidu A (2008) etal (2008) Epstein-Barr virus-induced gene 3 negatively regulates IL-17, IL-22 and RORgamma t. Eur J Immunol 38: 1204–1214. 18412165 10.1002/eji.200838145 PMC2989250

[6] Collison LW, Workman CJ, Kuo TT, Boyd K, Wang Y (2007) etal (2007) The inhibitory cytokine IL-35 contributes to regulatory T-cell function. Nature 450: 566–569. 18033300 10.1038/nature06306

[7] Collison LW, Chaturvedi V, Henderson AL, Giacomin PR, Guy C (2010) etal (2010) IL-35-mediated induction of a potent regulatory T cell population. Nat Immunol 11: 1093–1101. 20953201 10.1038/ni.1952 PMC3008395

[8] Wirtz S, Billmeier U, Mchedlidze T, Blumberg RS, Neurath MF (2011) Interleukin-35 mediates mucosal immune responses that protect against T-cell-dependent colitis. Gastroenterology 141: 1875–1886. 21820391 10.1053/j.gastro.2011.07.040 PMC3624892

[9] Liu JQ, Liu Z, Zhang X, Shi Y, Talebian F (2012) etal (2012) Increased Th17 and regulatory T cell responses in EBV-induced gene 3-deficient mice lead to marginally enhanced development of autoimmune encephalomyelitis. J Immunol 188: 3099–3106. 22387555 10.4049/jimmunol.1100106 PMC3311737

[10] Bettini M, Castellaw AH, Lennon GP, Burton AR, Vignali DA (2012) Prevention of autoimmune diabetes by ectopic pancreatic β-cell expression of interleukin-35. Diabetes 61: 1519–1526. 22427377 10.2337/db11-0784 PMC3357277

[11] Niedbala W, Wei XQ, Cai B, Hueber AJ, Leung BP (2007) etal (2007) IL-35 is a novel cytokine with therapeutic effects against collagen-induced arthritis through the expansion of regulatory T cells and suppression of Th17 cells. Eur J Immunol 37: 3021–3029. 17874423 10.1002/eji.200737810

[12] Huang CH, Loo EX, Kuo IC, Soh GH, Goh DL (2011) etal (2011) Airway inflammation and IgE production induced by dust mite allergen-specific memory/effector Th2 cell line can be effectively attenuated by IL-35. J Immunol 187: 462–471. 21613618 10.4049/jimmunol.1100259

[13] Klingenberg R, Gerdes N, Badeau RM, Gisterå A, Strodthoff D (2013) etal (2013) Depletion of FOXP3+ regulatory T cells promotes hypercholesterolemia and atherosclerosis. J Clin Invest 123: 1323–1334. 23426179 10.1172/JCI63891 PMC3582120

[14] Jia L, Zhu L, Wang JZ, Wang XJ, Chen JZ (2013) etal (2013) Methylation of FOXP3 in regulatory T cells is related to the severity of coronary artery disease. Atherosclerosis 228: 346–352. 23566804 10.1016/j.atherosclerosis.2013.01.027

[15] Lü CX, Xu RD, Cao M, Wang G, Yan FQ (2013) etal (2013) FOXP3 demethylation as a means of identifying quantitative defects in regulatory T cells in acute coronary syndrome. Atherosclerosis 229: 263–270. 23735638 10.1016/j.atherosclerosis.2013.05.007

[16] Meng X, Zhang K, Li J, Dong M, Yang J (2012) etal (2012) Statins induce the accumulation of regulatory T cells in atherosclerotic plaque. Mol Med 18: 598–605. 22331026 10.2119/molmed.2011.00471 PMC3388131

[17] Lin Y, Huang Y, Lu Z, Luo C, shi Y (2012) etal (2012) Decreased plasma IL-35 levels are related to the left ventricular ejection fraction in coronary artery diseases. PLoS One 7: e52490. 23285065 10.1371/journal.pone.0052490 PMC3528657

[18] Kempe S, Heinz P, Kokai E, Devergne O, Marx N (2009) etal (2009) Epstein-barr virus-induced gene-3 is expressed in human atheroma plaques. Am J Pathol 175: 440–447. 19556516 10.2353/ajpath.2009.080752 PMC2708829

[19] Minamino T, Komuro I, Kitakaze M (2010) Endoplasmic reticulum stress as a therapeutic target in cardiovascular disease. Circ Res 107: 1071–1082. 21030724 10.1161/CIRCRESAHA.110.227819

[20] Zhou AX, Tabas I (2013) The UPR in atherosclerosis. Semin Immunopathol 35: 321–332. 23553213 10.1007/s00281-013-0372-x PMC3967405

[21] Tabas I (2010) The role of endoplasmic reticulum stress in the progression of atherosclerosis. Circ Res 107: 839–850. 20884885 10.1161/CIRCRESAHA.110.224766 PMC2951143

[22] Scull CM, Tabas I (2011) Mechanisms of ER stress-induced apoptosis in atherosclerosis. Arterioscler Thromb Vasc Biol 31: 2792–2797. 22096099 10.1161/ATVBAHA.111.224881 PMC3220876

[23] McAlpine CS, Bowes AJ, Khan MI, Shi Y, Werstuck GH (2012) Endoplasmic reticulum stress and glycogen synthase kinase-3β activation in apolipoprotein E-deficient mouse models of accelerated atherosclerosis. Arterioscler Thromb Vasc Biol 32: 82–91. 21998135 10.1161/ATVBAHA.111.237941

[24] Li Y, Schwab RF, DeVries-Seimon T, Yao PM, Gerbod-Giannone MC (2005) etal (2005) Free cholesterol-loaded macrophages are an abundant source of tumor necrosis factor-alpha and interleukin-6: model of NF-kappaB- and map kinase-dependent inflammation in advanced atherosclerosis. J Biol Chem 280: 21763–21772. 15826936 10.1074/jbc.M501759200

[25] Gotoh T, Endo M, Oike Y (2011) Endoplasmic reticulum stress-related inflammation and cardiovascular diseases. Int J Inflam 2011: 259462. 21755026 10.4061/2011/259462 PMC3132612

[26] Luoma PV (2013) Elimination of endoplasmic reticulum stress and cardiovascular, type 2 diabetic, and other metabolic diseases. Ann Med 45: 194–202. 22928964 10.3109/07853890.2012.700116 PMC3581057

[27] Lenin R, Maria MS, Agrawal M, Balasubramanyam J, Mohan V (2012) etal (2012) Amelioration of glucolipotoxicity-induced endoplasmic reticulum stress by a “chemical chaperone” in human THP-1 monocytes. Exp Diabetes Res 2012: 356487. 22550476 10.1155/2012/356487 PMC3328920

[28] Devergne O, Birkenbach M, Kieff E (1997) Epstein-Barr virus-induced gene 3 and the p35 subunit of interleukin 12 form a novel heterodimeric hematopoietin. Proc Natl Acad Sci U S A 94: 12041–12046. 9342359 10.1073/pnas.94.22.12041 PMC23696

[29] Devergne O, Hummel M, Koeppen H, Le Beau MM, Nathanson EC (1996) etal (1996) A novel interleukin-12 p40-related protein induced by latent Epstein-Barr virus infection in B lymphocytes. J Virol 70: 1143–1153. 8551575 10.1128/jvi.70.2.1143-1153.1996 PMC189923

[30] Kim HJ, Jeong JS, Kim SR, Park SY, Chae HJ (2013) etal (2013) Inhibition of endoplasmic reticulum stress alleviates lipopolysaccharide-induced lung inflammation through modulation of NF-κB/HIF-1α signaling pathway. Sci Rep 3: 1142. 23359618 10.1038/srep01142 PMC3556596

[31] Wu ZH, Chen YQ, Zhao SP (2013) Simvastatin inhibits ox-LDL-induced inflammatory adipokines secretion via amelioration of ER stress in 3T3-L1 adipocyte. Biochem Biophys Res Commun 432: 365–369. 23376721 10.1016/j.bbrc.2013.01.094

[32] Ben Mosbah I, Alfany-Fernández I, Martel C, Zaouali MA, Bintanel-Morcillo M (2010) etal (2010) Endoplasmic reticulum stress inhibition protects steatotic and non-steatotic livers in partial hepatectomy under ischemia-reperfusion. Cell Death Dis 1: e52. 21364657 10.1038/cddis.2010.29 PMC3032561

[33] Li J, Wang JJ, Yu Q, Wang M, Zhang SX (2009) Endoplasmic reticulum stress is implicated in retinal inflammation and diabetic retinopathy. FEBS Lett 583: 1521–1527. 19364508 10.1016/j.febslet.2009.04.007 PMC2691649

[34] Kempe S, Heinz P, Kokai E, Devergne O, Marx N (2009) etal (2009) Epstein-barr virus-induced gene-3 is expressed in human atheroma plaques.Am J Pathol. 175: 440–447. 10.2353/ajpath.2009.080752 PMC2708829 19556516

[35] Gargalovic PS, Gharavi NM, Clark MJ, Pagnon J, Yang WP (2006) etal (2006) The unfolded protein response is an important regulator of inflammatory genes in endothelial cells. Arterioscler Thromb Vasc Biol 26: 2490–2496. 16931790 10.1161/01.ATV.0000242903.41158.a1

[36] Christen V, Meili N, Fent K (2013) Microcystin-LR induces endoplasmatic reticulum stress and leads to induction of NFκB, interferon-alpha, and tumor necrosis factor-alpha. Environ Sci Technol 47: 3378–3385. 23431999 10.1021/es304886y

